# Engineering CRISPR guide RNAs for programmable RNA sensors

**DOI:** 10.1042/BST20221486

**Published:** 2023-11-13

**Authors:** Yang Liu, Wei Liu, Baojun Wang

**Affiliations:** 1 MRC Laboratory of Molecular Biology (LMB), Francis Crick Avenue, Cambridge Biomedical Campus, Cambridge CB2 0QH, U.K.; 2 College of Chemical and Biological Engineering & Hangzhou Global Scientific and Technological Innovation Center, Zhejiang University, Hangzhou 310058, China; 3 Research Center for Biological Computation, Zhejiang Lab, Hangzhou 311100, China

**Keywords:** CRISPR, gRNA, programmability, RNA sensor

## Abstract

As the most valuable feature of the CRISPR system, the programmability based on Watson–Crick base pairing has been widely exploited in engineering RNA sensors. The base pairing in these systems offers a connection between the RNA of interest and the CRISPR effector, providing a highly specific mechanism for RNA detection both *in vivo* and *in vitro*. In the last decade, despite the many successful RNA sensing approaches developed during the era of CRISPR explosion, a deeper understanding of the characteristics of CRISPR systems and the continuous expansion of the CRISPR family members indicates that the CRISPR-based RNA sensor remains a promising area from which a variety of new functions and applications can be engineered. Here, we present a systematic overview of the various strategies of engineering CRISPR gRNA for programmable RNA detection with an aim to clarify the role of gRNA's programmability among the present limitations and future development of CRISPR-enabled RNA sensors.

## Introduction

The Clustered Regularly Interspaced Short Palindromic Repeats (CRISPR) system originated from the war between selfish genetic elements. Although it was thought to be a part of the bacterial immune mechanisms, unlike the early understanding, recent research shows that the CRISPR system also exists in phage and mobile genetic elements with incredible diversity and wide distribution [1–8], which has far exceeded the concept of the ‘immune mechanism of bacteria'. A typical CRISPR system is triggered by the complementary pairing of the guide RNA (gRNA) and their target DNA or RNA for identifying the friend and foe. The guide RNA can be either a single molecule or two separate parts. Taking the CRISPR/Cas9 system as an example of the type II CRISPR systems, the guide RNAs of Cas9 are naturally composed of a trans-activating CRISPR RNA (tracrRNA) and a CRISPR RNA (crRNA). The tracrRNA can bind to Cas9 as a handle while pairing with the crRNA, and the crRNA contains the spacer sequence directing the CRISPR complex to its target DNA. The crRNA and tracrRNA are generally fused as a single-guide RNA (sgRNA), which is widely used to achieve CRISPR functions for engineering purposes [9]. In the case of CRISPR/Cas12a, the representative of the type V CRISPR systems, its gRNA is naturally a single crRNA composed of a handle for Cas12a binding and a spacer for DNA targeting [10]. As the subsequent events of the DNA/RNA targeting, the activated CRISPR effectors of various mechanisms can degrade the target molecules, mediate DNA insertion, regulate transcription, cause cell death, or trigger an abortive phage infection response.

The programmable recognition of nucleic acid sequences is the fundamental capability of all CRISPR systems and the starting point of most engineering efforts on CRISPR systems. Yet, for RNA sensor design, the natural nucleic acid recognition ability of CRISPR systems is only applicable in limited scenarios, since the natural consequences directed by CRISPR systems may cause side effects or are incompatible with efficient reporting systems. Accordingly, instead of harnessing the inherent programmability of gRNA, researchers have reprogrammed different segments of gRNAs for RNA sensing. Here, by focusing on the programmability of different gRNA substructures, we summarize the various engineering strategies and the engineering logic of all CRISPR-enabled RNA sensors, including how the designs combine the natural functions of CRISPR, the application scenarios of RNA sensors and their effective reporting systems into one system. In addition, we aim to provide enlightening insights into the critical role of gRNA programmability in engineering CRISPR-based RNA sensors.

## Engineering RNA sensors based on the programmability of gRNA spacer sequences

The spacer refers to an inherent highly programmable sequence on the gRNA. It can guide the CRISPR effector to bind to the target nucleic acid (DNA or RNA), subsequently triggering the operation of the CRISPR effector. Therefore, a straightforward engineering logic of an RNA sensor is to utilize the natural nucleic acid recognition ability of the CRISPR system to detect the RNA of interest, which is also the principle of the first batch of CRISPR-enabled RNA sensors.

**Figure 1.**
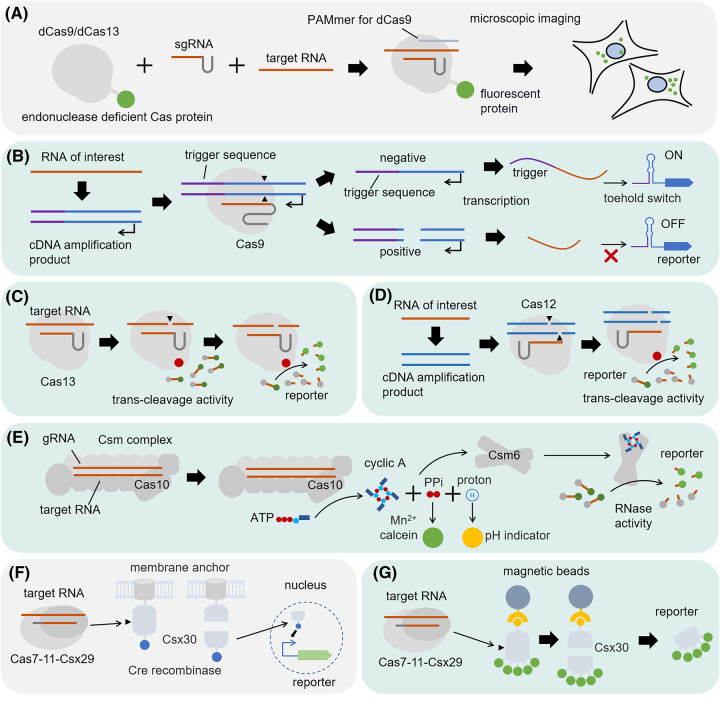
Design strategies of RNA sensors based on the programmability of gRNA spacer. Orange line represents the reprogrammed region (gRNA spacer, target RNA, or the RNA of interest), the light blue background represents applications *in vitro*, and the light grey background indicates applications *in vivo*. (**A**) CRISPR-mediated RNA tracking and imaging. (**B**) CRISPR/Cas9-assisted toehold-switch for SNP recognition of the RNA of interest. (**C**) RNA sensing via the trans-cleavage activity of Cas13. (**D**) RNA sensing via the trans-cleavage activity of Cas12. (**E**) CRISPR-mediated second messenger synthesis for RNA sensing. (**F**) CRISPR-mediated protein cleavage for RNA sensing *in vivo*. (**G**) CRISPR-mediated protein cleavage for RNA sensing *in vitro*.

An intuitive imagination is that if a programmable RNA-binding protein exists, the reporter molecule can be directly located and activated on the target RNA molecule. For example, the endonuclease deficient Cas9 (dCas9) or Cas13 (dCas13) protein can carry the fluorescent protein to the target RNA for generating an *in situ* fluorescent signal (Figure 1A). The Cas9 from *Streptococcus pyogenes* was discovered to have the ability to target single-stranded RNA in the presence of a short PAM-presenting DNA oligonucleotide (PAMmer). This mechanism was then employed for RNA detection and imaging in living cells [11,12]. Similarly, based on the RNA-guided RNA binding ability of the type VI CRISPR-effector Cas13, the dCas13 was also engineered by fusing with a fluorescent protein to track transcripts *in vivo* [13]. These applications all take advantage of the inherent programmability of the gRNA spacer to detect different RNA targets.

When applied in cell imaging, RNA sensors mainly display the spatial distribution of the target RNAs at the single-cell level. However, RNA sensing at the cell population level or *in vitro* often does not involve the spatial dimension. Therefore, non-imaging-related RNA detection requires more than simply recruiting the reporter molecule on the RNA of interest, where the level of the RNA of interest needs to be coupled with the output intensity of the reporter system.

For *in vitro* RNA sensing, the design was complicated at the beginning. The Zika virus outbreak that swept across the Americas from 2015 to 2016 coincided with an explosion in CRISPR research, which led to the earliest *in vitro* CRISPR RNA sensors. In 2016, Pardee et al. [14] reported the NASBA-CRISPR Cleavage (NASBACC) Assay, which utilizes CRISPR/Cas9 cleavage of the amplified complementary DNA (cDNA) of Zika virus genomic RNA to distinguish viral gene variants between different strains. Since the primary function of Cas9 is DNA recognition and cleavage, an additional reporter circuit under the control of a toehold switch was utilized to quantify the Cas9 activity by detecting the RNA transcribed from the Cas9-cleavaged cDNA (Figure 1B). A toehold switch is an RNA based switch composed of a switch RNA and a trigger RNA. Upon binding of the trigger RNA to the switch RNA, the buried ribosome binding site can be exposed, and translation of the downstream gene can be activated. When NASBACC happens, the DNA coding the full trigger RNA is cleaved, resulting in the transcription of a truncated trigger RNA which is unable to turn on the toehold switch and thus leaving the reporter off. In this case, due to the limitations of the mechanism, the CRISPR/Cas9 system played an auxiliary role in improving the specificity and resolution of the toehold switch-based RNA sensing.

The dramatic simplification of CRISPR-based RNA sensors *in vitro* comes from discovering the collateral catalytic activity of CRISPR effectors. The type VI CRISPR-effector Cas13a can non-specifically cleave collateral RNA when triggered by the gRNA-guided RNA targeting [15]. This mechanism is the key to CRISPR-mediated abortive infection response, which is toxic to cells. Coincidentally, it provides a superior mechanism for developing *in vitro* RNA sensors. When Cas13a targets and cleaves an RNA, it activates its non-specific RNA cleavage activity, subsequently digesting the RNA reporter molecules designed with a fluorescent dye and quencher, resulting in the generation of a fluorescent signal (Figure 1C) [16,17]. The RNA sensor was named Specific High-Sensitivity Enzymatic Reporter UnLOCKing (SHERLOCK). The multi-turnover trans-cleavage activity of Cas13a provides a way to couple the RNA target with the reporter and gives an additional amplification that improves the sensitivity of SHERLOCK. In addition, the above strategy allows the incorporation of a reverse transcription (RT) step to achieve amplification of the target RNA via the subsequent transcription of the cDNA. Based on the same principle of using DNA to transcribe large amounts of RNA, Cas12 or Cas14, which has collateral nuclease activity but targets DNA substrates, can also be used for RNA detection *in vitro* (Figure 1D) [18–21].

The ability of some CRISPR effectors to activate downstream accessory proteins, in addition to incidental nuclease activity, provides a promising mechanism. A typical case is the second messenger synthesis activity of type III CRISPR systems [22]. When activated by the target RNA, the Cas10 subunit of the CRISPR complex converts ATP into cyclic oligoA, which can activate RNase Csm6. Csm6 then digests RNA reporters and results in a corresponding fluorescent signal [22,23]. In addition, pyrophosphate and protons produced by Cas10 in catalyzing cyclic oligoA synthesis can also be utilized to design reporters for RNA sensing (Figure 1E) [23]. Recently, the accessory protease of the type III-E CRISPR systems was also engineered for RNA sensing. The protease Csx29 can be activated by Cas7–11–RNA complex and process the Csx30 protein which is an inhibitor of the transcription factor CASP-σ [24–26]. Interestingly, the RNA cleavage ability of the CRISPR complex is independent of its ability to activate the protease Csx29, making it possible to trigger the reporter without destroying the target RNA. By immobilizing Csx30 to either membrane proteins or magnetic beads, plus tethering alternative effectors instead of CASP-σ to Csx30, like the Cre recombinase or fluorescent labels, the CRISPR-associated endopeptidase has been engineered for RNA sensing both *in vivo* and *in vitro*. For the *in vivo* system, Cre recombinase is sequestered from the nucleus by fusing with a membrane anchor via a Csx30-derived linker. In the presence of target RNA, Csx30 is cleaved facilitating the liberation of Cre recombinase. The released Cre recombinase enters the nucleus and leads to green fluorescent protein (GFP) expression from a loxP-GFP reporter cassette (Figure 1F). For the *in vitro* sensor, the fluorescently labelled Csx30 releases fluorescence from bead-captured Csx30, only in the presence of RNA target (Figure 1G) [24].

In summary, for the above-described RNA sensors, the role of the CRISPR system is to target different RNA (or its amplified cDNA) via the inherent programmability of the gRNA spacer, which provides high specificity for RNA detection. In this class of RNA sensors, we see the excellent utilization of CRISPR collateral catalytic activity. The collateral catalytic activity mainly triggers the reporting system with a signal amplification function. That said, the highest sensitivity of the *in vitro* sensor still relies on the nucleic acid amplification steps instead of the latter step of collateral catalysis. Nevertheless, the Cas7–11–Csx29 system is an exception since it allows signal amplification *in vivo*.

## Engineering RNA sensors based on the programmability of gRNA scaffolds

The gRNA scaffold (handle) is often referred to as the remaining segment outside the spacer. This conserved region folds into a unique secondary structure and interacts with the CRISPR effector. The gRNA scaffolds vary widely in different types of CRISPR systems. For example, it can be a part of the CRISPR RNA (crRNA) or provided by a trans-bound RNA, like the trans-activating crRNA (tracrRNA) in the type II CRISPR system or the scoutRNA in type V CRISPR system [27,28].

The repeat region of crRNA usually constitutes gRNA scaffold, which is involved in many processes, including spacer acquisition, pre-crRNA processing, and efficient binding of crRNA to CRISPR effectors [29–31]. The above versatility makes the gRNA scaffold sequence conserved for a specific CRISPR system. However, a lot of evidence has suggested that the sequence of gRNA scaffolds is programmable regarding CRISPR effector functions alone [32].

**Figure 2.**
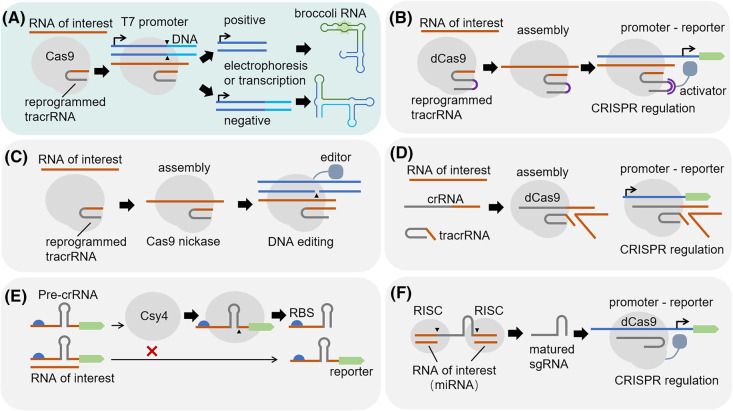
Design strategies of RNA sensors based on the programmability of gRNA scaffold. Orange line represents the reprogrammed region (gRNA scaffold and the RNA of interest), the light blue background represents applications *in vitro*, and the light grey background indicates applications *in vivo*. (**A**) Reprogrammed tracrRNA detects the RNA of interest *in vitro*. (**B**) Reprogrammed tracrRNA hijacks the RNA of interest for downstream genetic regulation. (**C**) Reprogrammed tracrRNA detects the RNA of interest and leads to DNA editing for transcriptional event recording. (**D**) RNA-mediated crRNA–tracrRNA assembly for downstream genetic regulation. (**E**) RNA interferes with the pre-crRNA processing. (**F**) miRNA-meditated pre-crRNA processing.

The programmability of the gRNA scaffold allows repurposing the RNA of interest as part of the gRNA to regulate the function of CRISPR system. Owing to the programmability of the crRNA–tracrRNA pairing, a new type of RNA sensor was developed [32,33]. Based on this design, the reprogrammed tracrRNA can hijack the RNA of interest to form a functional dual gRNA for CRISPR/Cas9 function. Since the RNA of interest is not the substrate of the CRISPR effector, the sensor will not destroy the RNA being detected nor significantly affect its original physiological function. The dual recognition is a unique advantage of this design since the sequence of the RNA of interest must pair with both the Cas9-targeted DNA and the reprogrammed tracrRNA for forming Cas9–gRNA–dsDNA complex and leading a sensing response [32]. The above mechanism thus provides a principle of engineering RNA sensors with the highest specificity to date. This design has also been employed for pathogenic RNA detection *in vitro*, especially during the SARS-Cov-2 pandemic, when two similar RNA sensors, AGATHA and LEOPARD, were independently developed to detect the viral RNA (Figure 2A) [32,33]. In both RNA sensors, the RNA of interest plays the role of crRNA and lead to DNA cleavage. For AGATHA, the complete target DNA can be transcribed into a non-functional RNA, while it can be transcribed into a functional broccoli RNA when cleaved. The broccoli RNA can then bind and activate the fluorophore, giving a fluorescent signal. Furthermore, by combining with the CRISPR activation device, which utilizes dCas9 and engineered gRNA to recruit the σ^54^-dependent transcriptional activator, the RNA of interest can trigger the expression of a reporter like GFP. It allows the detection of endogenous RNAs and the RNA-responsive transcriptional regulation *in vivo* (Figure 2B) [32]. This principle has also recently been combined with base editors to record transient transcriptional events in bacteria (Figure 2C) [34].

Another effective strategy is to utilize the RNA of interest to stabilize the crRNA–tracrRNA interaction. In this design, the crRNA–tracrRNA matching region was truncated and replaced with artificial end sequences. The artificial end sequences are designed to be partially complementary to the sequence of the RNA to be detected. Therefore, the RNA of interest can bind to the crRNA and tracrRNA through the artificial sequence, forming a trimeric RNA complex to function as the gRNA (Figure 2D). This strategy transformed the binary assembly form of crRNA–tracrRNA into a new type of ternary assembly form, based on which it allows the RNA of interest to stabilize the assembly of the gRNA in a programmable manner. The advantage of such design is that it decouples the input sequence of the RNA of interest from the gRNA spacer, in which way the independent programmability of the spacer is retained. Then the reporter circuit triggered by the CRISPR effector can be separately designed, which extends the simplicity and flexibility of the sensor output [35].

Additionally, a particular type of RNA sensor related to the gRNA scaffold programmability was based on pre-crRNA processing. For instance, utilizing the programmability of the flanking contexts of the Csy4 binding site (CBS), a pre-crRNA can be designed as the reporter. When the RNA of interest specifically binds to the CBS flanking regions and prevents pre-crRNA cleavage by Csy4, the reporter translation is positively regulated (Figure 2E). This mechanism works at the level of pre-crRNA processing via the programmable region outside the spacer, which is broadly associated with the gRNA scaffold [36]. An alternative is adding additional sequences to a sgRNA of Cas9 to generate artificial pre-gRNA. As a result, the miRNA or siRNA-mediated RNA cleavage can then generate mature sgRNA and activate CRISPR function, which has been used for microRNA detection *in vivo* (Figure 2F) [37]. Instead of utilizing the inherent programmability of the gRNA scaffold, the mechanism of this type of RNA sensors relies mainly on RNA processing and only partially on the function of CRISPR protein.

Compared with gRNA-spacer-based RNA sensor, the advantage of a gRNA-scaffold-based sensor is that the RNA of interest is no longer the target of the CRISPR effector. Thereby, the native functions of CRISPR effectors can be utilized for downstream engineering. In principle, the gRNA-scaffold-based sensors can be compatible with all currently known CRISPR applications.

## Engineering RNA sensors based on RNA strand displacement

Although the strand displacement does not always rely on the inherent programmability of native gRNAs, it is undoubtedly a great attempt to endow gRNA with artificial programmability if the riboswitches can be seen as additional parts of gRNA. In this strategy, when the RNA of interest binds to the initial gRNA structure and subsequently alters the RNA folding, the gRNA can then be activated or inactivated. This strategy is inspired by combining the toehold switch riboregulators and the CRISPR gRNAs in synthetic biology [38–41]. As mentioned above, the toehold switch was initially designed for translational regulation by controlling the interaction of the trigger and switch RNAs. With similar RNA interaction principle, it can be further expanded to use the RNA of interest as the trigger RNA to control the functional status of the CRISPR gRNA by altering its secondary structure.

**Figure 3.**
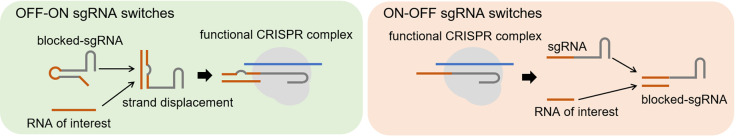
Design strategy of the OFF-ON sgRNA switches (left), and the ON-OFF sgRNA switches (right).

One category is the OFF-ON sgRNA switches. The gRNAs with additional structures fold into a non-functional conformation by default. When an RNA of interest binds to the double strand or flanking region of the gRNA, the released RNA strand can refold into a functional structure for gRNA function (Figure 3). Almost all essential segments of a gRNA can be functionally locked by an artificially designed complementary strand and then released during a subsequent strand displacement to restore its function [42,43]. When the sequence of the replaced RNA strand is programmable (such as a spacer segment or flanking region), the sgRNA switches can be used to detect different RNAs. This strategy has been used in *E. coli* and mammalian cells to detect small RNAs and mRNAs [38,44–48].

Another category is the ON-OFF sgRNA switches. The design principle is to add harmless programmable flanking segments to the essential structure of the gRNA. When the RNA of interest binds to these flanking segments, the function of the gRNA will be destroyed, thereby modulating the operation of the CRISPR effector (Figure 3) [39].

In summary, gRNA under strand displacement control is a mature tool that either takes advantage of the inherent programmability of the gRNA spacer region or expands the gRNA's programmability by incorporating customized programmable flanking sequences for detecting different RNA molecules. Instead of only mining the inherent programmability of gRNA, this strategy can increase the space of programmability of gRNAs through artificial design.

## Discussion

The unique RNA–protein association of CRISPR systems provides an exciting platform for developing various programmable bioengineering tools. As described above, the programmability of gRNAs has been explored thoroughly, involving spacer, scaffold, and additional flanking segments. The strategies involve not only the reprogramming at the level of primary structure but also secondary structure. Previous literature has stated the advantages of employing programmability of CRISPR-based devices. For instance, programmable devices with a unified mechanism can simplify the engineering process and improve the predictability of their functions; the programmability also supports highly orthogonal biological functions to enhance the robustness of devices and support multiplexed and scalable engineering [39,49–54].

However, the programmability of gRNA is not unlimited. Regarding tools that utilize spacer programmability for RNA detection, CRISPR effectors show sequence preference when targeting nucleic acids, such as the requirement for protospacer adjacent motif (PAM) or protospacer flanking sites (PFS). The spacer sequences also affect the folding of the gRNA, the efficiency of CRISPR binding or cleavage, and the probability of off-target activity [32,55,56]. Thanks to the enormous popularity of the CRISPR system, substantial engineering efforts have been made to address such issues. For example, Cas protein variants with relaxed PAM sequence requirements have been developed, as well as the dry lab tools to predict efficient spacer sequences [55–62]. In contrast, the programmability of gRNA scaffolds needs to be studied further. In the existing cases, the CRISPR effectors showed sequence preference for the programmable regions in gRNA or pre-crRNA [32,33,35,36]. As a specific example, in the CRISPR/Cas9 system, the different sequences of the pairing regions between artificial tracrRNAs and corresponding non-canonical crRNAs can lead to various DNA binding affinities [32]. For artificial gRNAs involving strand displacement, though the coupling of the extra programmable parts with the inherently programmable regions of the gRNA has been avoided, the potentially harmful RNA secondary structure still restricts the programmability of RNA sensors to some extent [43]. In such cases, although the selection of the RNA targeting site is not limited by the sequence of the gRNA spacer or handle, scientists further employ algorithms such as NUPACK to avoid the presence of intrinsic secondary structure of the RNA of interest [39,45].

The guiding principle for selecting target sites will be critical for all RNA sensor users. However, this is related to more than just the programmability of gRNAs. Variation in detection efficiency resulted from different target sites of the RNA of interest is a common issue for CRISPR-based RNA sensors, riboregulators, and the base editing-based RNA sensor that has been developed recently [63–65]. Our previous study has shown that the difference in detection efficiency between different target sites of the RNA of interest could be owing to multiple factors, which is not easy to be predicted accurately [32]. Pelea et al. [43] proposed that the experience from designing RNA-targeted hybridization probes may be used to predict the availability of effective target sites of the RNA to be detected. The experience from designing RNA probes could be worth exploring further. However, it remains unclear whether the interaction mode and binding affinity between CRISPR effectors and trigger RNAs is similar to that of RNA probes. Another promising approach is to use machine learning to address such issues. Machine learning is good at inferring causal relationships based on a given data set. For RNA sensors, it may predict the availability of a specific target site and the corresponding sensing output. For example, the machine learning method, linear discriminant analysis (LDA), was used to reveal the programmability of sgRNA scaffolds in the CRISPR/Cas9 system. Since the RNA pairing region in the sgRNA scaffold has been engineered to design RNA sensors, LDA is also suitable for searching efficient RNA target sites [66]. Nevertheless, it is worth noting that researchers may risk ignoring differences between artificially designed libraries and real-world situations by attempting to train the machine learning model with data generated from the artificial libraries. This is because the standardized RNA molecules under ideal reaction conditions cannot represent the variable RNA molecules in more complicated environments *in vivo*. Without practical evidence, results obtained from artificial sequence libraries cannot be generalized for all applications [32].

In summary, the programmability of gRNAs has been exploited for RNA sensors in various scenarios with diverse mechanisms, designs and characteristics. When users are facing many different methods and strategies, it is justifiable that it is difficult to make a choice. However, due to a lack of uniform experimental conditions, it is challenging to fairly compare the performance of the various types of RNA sensors. The most common challenge for biosensor engineers is to compare the strength, sensitivity, or dynamic range of different sensors and only a few studies out of specific application scenarios have been dedicated to test them. Many efforts remain to be made to address the limitations of CRISPR-enabled RNA sensors, such as to improve the ability to predict available target sites on the RNA of interest, to understand the orthogonality of RNA sensors to the host cells, to set up standards for describing and comparing the performance of different RNA sensors, and to mine more programmable CRISPR systems for engineering. Among the aforementioned efforts, the programmability of gRNAs is a critical factor for engineering but needs to be considered together with the other factors to generate impactful outcomes.

## Perspectives

- The programmability of gRNA lays the foundation of CRISPR-based RNA sensors, which have been exploited for various high-value applications, including imaging, monitoring, recording, quantitative measurement of RNA transcripts *in vivo*, or RNA detection *in vitro*.
- Further improving the programmability of gRNA is a mean to optimize CRISPR-based RNA sensors. However, it is only one of the critical factors determining their performance and usability.
- Performance predictability would be vital for future developments in CRISPR-enabled RNA sensors, for which dry lab techniques like machine learning will be valuable tools to contribute.

## Open Access

Open access for this article was enabled by the participation of University of Cambridge in an all-inclusive *Read & Publish* agreement with Portland Press and the Biochemical Society under a transformative agreement with JISC.

## Abbreviations

CBS: Csy4 binding site
LDA: linear discriminant analysis
NASBACC: NASBA-CRISPR Cleavage
PAM: protospacer adjacent motif
SHERLOCK: Specific High-Sensitivity Enzymatic Reporter UnLOCKing
